# Seasonal temperature acclimatization in a semi-fossorial mammal and the role of burrows as thermal refuges

**DOI:** 10.7717/peerj.4511

**Published:** 2018-03-16

**Authors:** Charlotte R. Milling, Janet L. Rachlow, Mark A. Chappell, Meghan J. Camp, Timothy R. Johnson, Lisa A. Shipley, David R. Paul, Jennifer S. Forbey

**Affiliations:** 1 Department of Fish and Wildlife Sciences, University of Idaho, Moscow, ID, USA; 2 School of Environment and Natural Resources, Ohio State University, Columbus, OH, USA; 3 Department of Evolution, Ecology, and Organismal Biology, University of California, Riverside, Riverside, CA, USA; 4 School of the Environment, Washington State University, Pullman, WA, USA; 5 Department of Statistical Science, University of Idaho, Moscow, ID, USA; 6 Department of Movement Sciences, University of Idaho, Moscow, ID, USA; 7 Department of Biology, Boise State University, Boise, ID, USA

**Keywords:** Burrow, *Brachylagus idahoensis*, Respirometry, Thermoregulatory costs, Pygmy rabbit, Thermal refuge

## Abstract

Small mammals in habitats with strong seasonal variation in the thermal environment often exhibit physiological and behavioral adaptations for coping with thermal extremes and reducing thermoregulatory costs. Burrows are especially important for providing thermal refuge when above-ground temperatures require high regulatory costs (e.g., water or energy) or exceed the physiological tolerances of an organism. Our objective was to explore the role of burrows as thermal refuges for a small endotherm, the pygmy rabbit (*Brachylagus idahoensis*), during the summer and winter by quantifying energetic costs associated with resting above and below ground. We used indirect calorimetry to determine the relationship between energy expenditure and ambient temperature over a range of temperatures that pygmy rabbits experience in their natural habitat. We also measured the temperature of above- and below-ground rest sites used by pygmy rabbits in eastern Idaho, USA, during summer and winter and estimated the seasonal thermoregulatory costs of resting in the two microsites. Although pygmy rabbits demonstrated seasonal physiological acclimatization, the burrow was an important thermal refuge, especially in winter. Thermoregulatory costs were lower inside the burrow than in above-ground rest sites for more than 50% of the winter season. In contrast, thermal heterogeneity provided by above-ground rest sites during summer reduced the role of burrows as a thermal refuge during all but the hottest periods of the afternoon. Our findings contribute to an understanding of the ecology of small mammals in seasonal environments and demonstrate the importance of burrows as thermal refuge for pygmy rabbits.

## Introduction

In mid- and high-latitudes, the thermal environment can vary substantially across spatial and temporal scales, such that ambient conditions can be energetically challenging for animals. For example, black-capped chickadees (*Parus atricapillus;* Cooper & Swanson, 1994), least weasels (*Mustela nivalis;* Casey & Casey, 1979), and red squirrels (*Tamiasciurus hudsonicus;* Irving, Krog & Monson, 1955) are north temperate endotherms that experience thermal conditions that can impose high thermoregulatory costs during winter. Adaptations to seasonal climate extremes include hibernation or torpor (Geiser & Ruf, 1995), physiological acclimatization (i.e., seasonal changes in insulation or temperature-dependent energy expenditure; Hinds, 1977; Heldmaier & Steinlechner, 1981; Rogowitz, 1990; Sheriff et al., 2009), and behavioral thermoregulation (i.e., temperature-dependent selection of habitats or use of thermal refuges; Sharpe & Van Horne, 1999; Walsberg, 2000). For small endotherms that do not migrate or hibernate, winter can be especially challenging because scarce food resources might not compensate for the increased energy demands of thermoregulation. Thus, changes in space use in response to the thermal environment may allow animals to persist in habitats with unfavorable thermal conditions while minimizing energy expenditure (Huey, 1991; Williams, Tieleman & Shobrak, 1999). For many small endotherms, burrows provide thermal refuge critical for maintaining homeothermy and reducing thermoregulatory costs during periods of extreme cold (Chappell, 1981; Harlow, 1981) and heat (Williams, Tieleman & Shobrak, 1999; Walsberg, 2000; Long, Martin & Barnes, 2005; Zungu, Brown & Downs, 2013).

An understanding of the relationship between temperature and physiology, and how that relationship changes seasonally, can help define the thermal roles of habitat features such as burrows. For endotherms, the thermoneutral zone (TNZ) is the range of ambient temperatures over which an animal can maintain body temperature (*T*_b_) through changes in posture, fur or feather orientation, and blood flow at the periphery (McNab, 2002; Lighton, 2008) without changes in metabolic rate. The TNZ is bounded on the warm end by the upper critical temperature (*T*_uc_) and on the cool end by the lower critical temperature (*T*_lc_). As ambient temperature increases above the *T*_uc_, resting metabolic rate (RMR) increases due to evaporative cooling (i.e., sweating or panting), and as temperature decreases below the *T*_lc_, RMR increases to maintain *T*_b_ (McNab, 2002). Energy expenditure within the TNZ is known as thermoneutral or minimal resting metabolic rate (RMR_T_) if the animal is inactive. The energetic costs of thermoregulation over the range of temperatures animals experience in their environment can be estimated using data on the TNZ, the RMR_T_, and the relationship between RMR and temperatures below the *T*_lc_ and above the *T*_uc_ (Weathers & Sullivan, 1993).

Seasonal physiological acclimatization allows endotherms to reduce thermoregulatory costs imposed by seasonally variable climates. Increased insulation from fat or pelage can reduce the *T*_lc_ in winter relative to summer and lessen the effect of temperature on RMR below the *T*_lc_ (Rogowitz, 1990; Holloway & Geiser, 2001), resulting in improved energy conservation at cold temperatures. For example, winter-acclimatized snowshoe hares had denser and longer fur, which contributed to a lower *T*_lc_ and thermal conductance, and helped reduce thermoregulatory costs at a time when food quantity and quality were low (Sheriff et al., 2009). A lower RMR_T_ in cold-acclimatized animals also would be adaptive in environments where food resources are limited (Holloway & Geiser, 2001; Sheriff et al., 2009), and a lower RMR_T_ in summer-acclimatized animals would be advantageous for conserving energy and water and minimizing heat production in hot desert environments (Hinds, 1973; Lovegrove, 1987). Some hot-acclimatized endotherms are able to elevate their *T*_uc_ (Hinds, 1973, 1977; Zervanos, 1975), which allows them to maintain homeothermy at higher temperatures without having to rely on evaporative cooling. Thermoregulatory costs can dominate the energy budgets of small mammals in strongly seasonal environments (Speakman, 1997), and seasonal acclimatization strategies and selective use of microhabitats can help reduce the energetic requirements. The costs of thermoregulating in different habitats can affect fitness either incrementally (e.g., by influencing resource acquisition behaviors) or absolutely (e.g., by increasing risk of predation; Huey, 1991; Humphries & Umbanhowar, 2007).

Our goal was to understand how a small endotherm, the pygmy rabbit (*Brachylagus idahoensis*), uses the refuge of a burrow to address thermal challenges in a strongly seasonal environment. Pygmy rabbits are endemic to the arid sagebrush habitats in the Intermountain West (Green & Flinders, 1980), which is characterized by extreme diurnal and seasonal fluctuations in temperature (Wise, 2012). Winter temperatures can be as low as −40 °C, and summer temperatures can reach 45 °C (Knapp, 1997). Their small size (400–500 g) and high surface area to volume ratio should engender high costs of regulatory heat production in typical winter temperatures, however, because their winter diet can be comprised nearly entirely of sagebrush (Shipley et al., 2006), they are not confronted with the challenge of food scarcity over winter. Furthermore, pygmy rabbits do not hibernate or huddle and leporids in general do not have high levels of body fat for insulation or energy reserves (Whittaker & Thomas, 1983). Nevertheless, pygmy rabbits can be active at all times of the day throughout the year (Larrucea & Brussard, 2009; Lee et al., 2010; Milling et al., 2017). Unlike most lagomorphs in North America, pygmy rabbits are obligate burrowers, and the ameliorated temperatures within burrows likely provide refuge from above-ground thermal conditions (Pike & Mitchell, 2013). However, burrow use by pygmy rabbits is highly variable among seasons (Larrucea & Brussard, 2009; Lee et al., 2010) and individuals (C. R. Milling, 2015, unpublished data), and may be influenced by a number of other factors including reproductive status and perception of predation risk (Rachlow, Sanchez & Estes-Zumpf, 2005; Camp et al., 2012). These unique traits make pygmy rabbits a compelling model organism to evaluate the functional roles of burrows as thermal refuges during summer and winter.

To evaluate the role of burrows for thermoregulation by pygmy rabbits, we had three objectives: (1) measure the relationship between temperature and oxygen consumption during summer and winter using indirect calorimetry; (2) measure the thermal environment within burrows and at above-ground rest sites near burrow systems known to be used by pygmy rabbits during summer and winter; and (3) combine these datasets to estimate the approximate thermoregulatory costs of resting in burrows and above-ground microsites during both seasons. We hypothesized that the relationship between energy expenditure and temperature would vary from summer to winter, reflecting seasonal physiological acclimatization (i.e., changes in insulation) to prevailing thermal conditions, such that thermoregulatory costs would be higher in summer than winter at temperatures below the TNZ. Because burrow use by pygmy rabbits is poorly understood and can be influenced by a variety of factors (e.g., predation risk, reproduction, and thermal constraints), we did not articulate specific hypotheses about how burrow use should differ with thermal conditions. Instead, we sought to quantify the costs associated with thermoregulating inside the burrow relative to above-ground rest sites in both seasons as a first step towards evaluating the costs and benefits of burrow use. However, we did expect that the burrow would serve as a thermal refuge for a greater proportion of time during winter than summer because temperatures can remain well below freezing for extended periods during the winter. We also expected that the thermoregulatory costs associated with resting in the burrow would be lower than above-ground rest sites overnight in winter and during mid-day in summer. Elucidating the role of burrows as thermal refuges for pygmy rabbits contributes to an understanding of the species’ thermal ecology and the functional role of habitat in mitigating physiologically stressful conditions.

## Methods

### Thermal physiology

We evaluated RMR as rates of oxygen consumption by adult pygmy rabbits captured in east-central and south-central Idaho and in southwestern Montana, USA (Idaho Department of Fish and Game Scientific Collection Permits #010813 and #100310; Montana Fish, Wildlife, and Parks Scientific Collection Permit #2014-062). We maintained the animals in captivity at the Small Mammal Research Facility at Washington State University. Animals were housed individually in 1.8 × 1.2 m mesh pens lined with pine shavings inside of a barn with a roof and partial walls. This arrangement exposed the rabbits to ambient temperatures but protected them from direct solar radiation, wind, and precipitation. Cages had corrugated pipe and nest boxes for enrichment and refuge. Food (Purina Professional Rabbit Chow, Purina Mills, St. Louis, MO, USA) and water were available ad libitum. Daily maximum and minimum ambient temperatures were recorded in the facility using a digital thermometer (model number 00613CASB; Chaney Instrument Co., Lake Geneva, WI, USA) during winter (4 January–17 March, 2016) and summer (13 June–7 July, 2016) trial periods. Mean daytime ambient high temperatures in the facility during winter were 7.8 °C (sd = 3.4, range = 2.2–15 °C) and mean lows were 1.1 °C (sd = 2.7, range = −4.4–6.6 °C). During summer, mean daytime ambient highs were 35.5 °C (sd = 6.7, range = 23.3–45.0 °C) and mean lows were 8.4 °C (sd = 2.6, range = 3.3–12.2 °C).

We measured rates of oxygen consumption ($\dot V{{\rm{O}}_2}$, ml O_2_/min) during winter (5 January–13 March, 2016) and summer (13 June–7 July, 2016) across a range of temperatures typical of natural habitats. During winter, we evaluated $\dot V{{\rm{O}}_2}$ at seven temperatures ranging from approximately −5 to 25 °C and in the summer at six temperatures ranging from approximately 5 to 30 °C. Trials were conducted between 0800 and 1800 h and animals were only exposed to one treatment temperature per day (to the extent that we were able, animals were not subjected to trials on successive days). We did not fast the animals prior to treatment because pygmy rabbits produce and consume cecal pellets and might not be truly post-absorptive, and Katzner, Parker & Harlow (1997) did not detect an effect of fasting on RMR of pygmy rabbits. Animals were weighed before each trial and placed in an airtight plexiglass metabolic chamber (volume = 4,500 cm^3^). Because body heat can influence the internal temperature of the metabolic chamber, we measured temperature inside the chamber using two iButtons® (*T*_c_; model number DS1921G; Maxim Integrated, San Jose, CA, USA) positioned on diagonally opposed corners. For all temperatures ≥0 °C, the metabolic chamber was placed inside a temperature controlled environmental cabinet. For the −5 °C winter temperature trials, the chamber was placed inside of a small freezer. We used a wireless infra-red camera (model number NC223W-IR; Shenzhen Anbash Technology, Shenzhen, China) to monitor the activity and welfare of animals.

We used a pushed flow-through respirometry system to measure $\dot V{{\rm{O}}_2}$ for 2 h, with the first hour allowing acclimation to the trial temperature and the second hour comprising the sampling interval. Water vapor was removed from room air using a Drierite column (W.A. Hammond Drierite Co., Xenia, OH, USA), and the dried air was forced into the metabolic chamber at a controlled flow rate of 4000 mL/min using a mass flow controller (model 32907-71; Cole Parmer, Vernon Hills, IL, USA). Excurrent air was subsampled, scrubbed of moisture and CO_2_ using Drierite and indicating soda lime, and pushed into a fuel cell oxygen analyzer (FC-10; Sable Systems, North Las Vegas, NV, USA). Flow rate into the chamber and oxygen concentrations were averaged over 5 s intervals, converted to digital signal by an A–D converter (UI-2; Sable Systems), and recorded to a laptop using Warthog LabHelper software (Chappell, 2016b; http://www.warthog.ucr.edu). We collected baseline concentrations of room air for 3–5 min at the start of the trial and approximately every 40 min thereafter to correct for drift in the oxygen analyzer. We used Warthog LabAnalyst software to fit a regression to baseline oxygen concentrations and corrected oxygen concentrations accordingly. The $\dot V{{\rm{O}}_2}$ was calculated by LabAnalyst (Chappell, 2016a) as:

$$\dot V{{\rm{O}}_2} = ({{\rm{F}}_{\rm{i}}}{{\rm{O}}_{\rm{2}}} - {{\rm{F}}_{\rm{e}}}{{\rm{O}}_{\rm{2}}})*{\rm{FR}}/(1 - {{\rm{F}}_{\rm{e}}}{{\rm{O}}_{\rm{2}}})$$

where FR is the incurrent mass flow rate scrubbed of water vapor and CO_2_; F_i_O_2_ is the fractional oxygen concentration in dry, CO_2_-free air (0.2095); and F_e_O_2_ is the fractional oxygen concentration of excurrent air scrubbed of water vapor and CO_2_. Data were visually inspected, and mean values of oxygen consumption were obtained when $\dot V{{\rm{O}}_2}$ was low and stable, reflecting RMR. Precision of the oxygen analyzer was validated via ethanol combustion (Lighton, 2008). We estimated whole body thermal conductance (*C*; mL O_2_ h^−1^ °C^−1^; multiply by 0.00558 for watts/°C) for each animal at the coldest trial temperature in summer and winter according to the Irving–Scholander model, $C = \dot V{{\rm{O}}_2}/({T_{\rm{b}}} - {T_{\rm{c}}})$, using previously reported values of winter *T*_b_ for pygmy rabbits (Katzner, Parker & Harlow, 1997). Although body temperature can vary seasonally, several species of lagomorph maintain constant body temperature year-round (Hinds, 1973, 1977; Sheriff et al., 2009). In the absence of evidence to the contrary, we assumed the same *T*_b_ for summer- and winter-acclimatized pygmy rabbits. All animal protocols were approved by the Institutional Animal Care and Use Committees at University of Idaho (Protocols #2012-23 and #2015-12) and Washington State University (Protocol #04398-011), and they were in accordance with guidelines for the use of wild mammals in research published by the American Society of Mammalogists (Sikes & Animal Care and Use Committee of the American Society of Mammalogists, 2016).

### Microsite temperature

We evaluated the thermal environment in above-ground microsites and burrows available to pygmy rabbits in sagebrush steppe habitat in the Lemhi Valley of east-central Idaho, USA. The valley is high-desert shrub-steppe (elevation = 1,880–2,020 masl) and receives on average <25 cm precipitation annually (Western Regional Climate Center, 2016), most of which falls as rain during late spring. Average temperatures range from a daytime low of −15.7 °C to a high of −1.2 °C in January and 5.4–29 °C in July (Western Regional Climate Center, 2016). The study site is characterized by mounded microtopography known as mima-mounds (Tullis, 1995). These mounds tend to have deeper soils and support taller shrubs than the surrounding matrix, and they are where most pygmy rabbit burrow systems are located. Wyoming big sagebrush (*Artemisia tridentata wyomingensis*) is the dominant shrub species, with black sagebrush (*A. nova*), three-tip sagebrush (*A. tripartita*), green rabbitbrush (*Chrysothamnus viscidiflorus*), and rubber rabbitbrush (*Ericameria nauseosa*) occurring less frequently. The matrix between clusters of sagebrush supports a highly variable mix of low-growing shrubs, forbs, and bare ground in the lowest elevations, and more continuous grass and forb cover at higher elevations.

**Figure 1:**
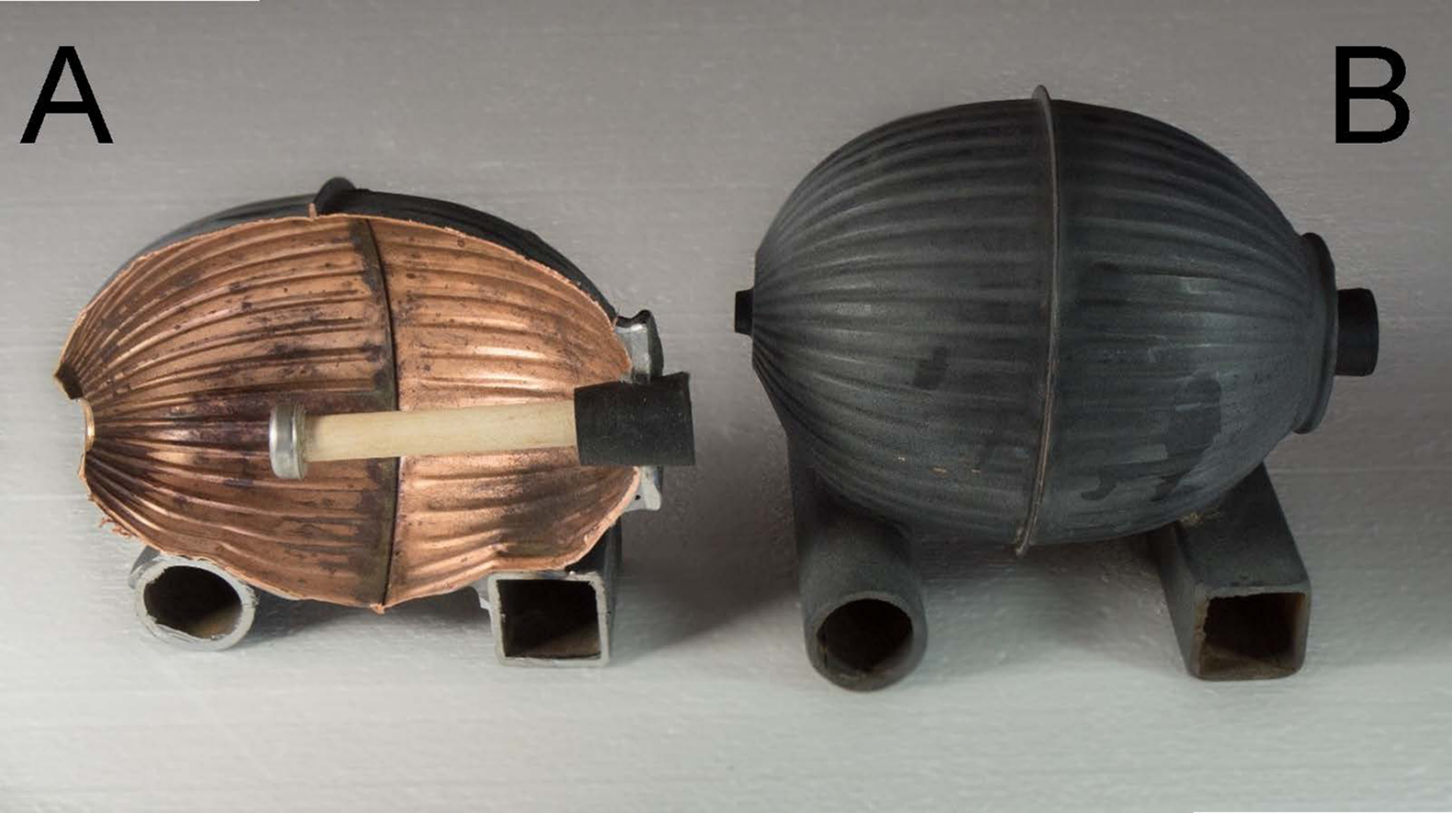
Cross-section of an operative temperature model (A) and an intact model (B) used to characterize the thermal environment in pygmy rabbit (*Brachylagus idahoensis*) habitat in eastern Idaho, USA. Photo credit: Charles Peterson.

We used operative environmental temperature (*T*_e_) to characterize the thermal environment of above-ground sites available to pygmy rabbits (Bakken, 1980; Coleman & Downs, 2010). *T*_e_ integrates heat transfer from radiative, conductive, and convective sources into a single index that specifies the equilibrium temperature an animal lacking metabolic heat production or evaporative heat loss would attain in a given combination of air temperature, wind, and sunlight. To measure *T*_e_, we built 10 models of the approximate size and shape of a resting pygmy rabbit (Bakken, 1992). Models were hollow copper ovoids (12.7 × 10.2 cm) painted a matte dark gray. Copper models have been shown to more precisely measure the above-ground thermal environment experienced by small, diurnal mammals and capture the thermal heterogeneity of habitat better than direct measures of temperature (Coleman & Downs, 2010). We attached two 8 cm segments of pipe to the bottom of the ovoids to prevent the devices from resting directly on the ground and to anchor them to the substrate. A calibrated iButton® attached to a wooden dowel was inserted into one end of each model and sealed in place by a rubber stopper (Fig. 1). The iButton® recorded temperature every 30 min for one month (31 days) in the winter (20 January–19 February, 2015) and summer (5 July–4 August, 2015) to capture the coldest and hottest times of the year in the valley.

Pygmy rabbit burrow systems were surveyed on foot during October 2014 according to methods described in Price & Rachlow (2011). Ten active burrow systems were randomly selected for temperature monitoring using ArcMAP 10.2 (ESRI, Redlands, CA, USA). Operative temperature models were placed at random locations on active burrow systems by identifying a random direction (cardinal or intercardinal) and distance (0–3 m, at 0.5 m increments) from the center of the mima mound. If the random point landed in a location where a pygmy rabbit could not reasonably be expected to rest (e.g., above ground on the trunk of a sagebrush plant), the model was placed adjacent to the obstruction as close to the random point as possible. The mean diameter of mima mounds at our study site was 10.6 m (range: 6.6–15.0 m; Parsons et al., 2016), and an entire burrow system was typically encompassed by the boundary of discrete mima mounds (McMahon et al., 2017). Pygmy rabbits at this site predominantly use mima mounds with burrow systems (McMahon et al., 2017) and rest close to burrow openings (Milling et al., 2017). Thus, the orientation of *T*_e_ models on the mound allowed us to capture a range of above-ground microclimates available to pygmy rabbits, and although the *T*_e_ model locations do not represent rest sites where rabbits were observed, they were located on active burrow systems and we often noted fresh fecal pellets within 0.5 m of the sensor locations. We identified 10 additional active burrow systems at which we monitored temperature within burrows; we included only burrows with a minimum of two openings because pygmy rabbits typically construct burrow systems with multiple openings (Green & Flinders, 1980). Because the thermal environment inside the burrow is not directly influenced by short wave radiation, and we assumed minimal influence of convection, we measured burrow temperature using Onset Stowaway TBI32 Tidbit temperature loggers (hereafter, tidbit; Onset Computer Corp., Bourne, MA, USA). We deployed one tidbit to a depth of 1 m (the average maximum depth of a pygmy rabbit burrow; Green & Flinders, 1980) within a randomly selected opening for each burrow by attaching the tidbit to stiff wire nailed at the burrow entrance. This inhibited removal by animals. Tidbits recorded temperature every 10 min for one month in winter (20 January–19 February, 2015) and summer (5 July–4 August, 2015). Of 10 tidbits deployed, one tidbit failed during winter, resulting in nine winter-monitored burrows, and two tidbits failed during summer, resulting in eight summer-monitored burrows. We calculated the mean temperature recorded by all tidbits and also by all operative temperature models per hour to estimate below- and above-ground temperatures with the same temporal resolution.

### Statistical analysis

Respirometry data were analyzed using mixed-effects segmented regression to evaluate the relationship between $\dot V{{\rm{O}}_2}$ and temperature during summer and winter (Gallant & Fuller, 1973). We used season and *T*_c_ as predictor variables with body mass as a covariate, and we included a random effect for individual identity. The model parameterizes the segments of the relationship between $\dot V{{\rm{O}}_2}$ and temperature below and within the TNZ, determines the breakpoint between segments, and estimates the influence of season and mass on the height of the function. From this output, we quantified the slope of the relationship below the *T*_lc_ in both seasons, determined the value of the *T*_lc_ (the breakpoint), and estimated summer RMR_T_. We tested for a seasonal difference in thermal conductance using a mixed-effects model with fixed effects for season and mass and a random effect for individual identity. Analyses were conducted using the “nlme” and “lme4” packages in R (R Core Team, 2016; Bates et al., 2015; Pinheiro et al., 2016), and results were deemed significant if *p* < 0.05 or if a 95% confidence interval on the parameter estimate did not capture zero. Values are reported as mean ± SE, unless otherwise specified.

We used results of the regression analyses to estimate approximate seasonal energetic costs of thermoregulation for animals at rest in burrows and in above-ground microsites. We set mass to the mean of our study animals and populated our temperature predictor variable (*T*_c_) using measurements of mean *T*_e_ and mean burrow temperature. We calculated the proportion of time in each season that the burrow could serve as a thermal refuge for a resting pygmy rabbit. In winter, this was defined as the amount of time that mean *T*_e_ < *T*_lc_, but the burrow was warmer than mean *T*_e_ and therefore had lower associated thermoregulatory costs. In summer, it was calculated as the amount of time that *T*_e_ > 35 °C (the average *T*_uc_ of pygmy rabbit-size eutherian mammals; see Araujo et al., 2013) and the burrow was cooler than *T*_e_. Additionally, we calculated the amount of energy (in kJ, where 20.1 J is equal to 1 mL O_2_; Schmidt-Nielsen, 1997) required to thermoregulate for the entire month in burrows exclusively, in above-ground microsites exclusively, and in a combination of the two habitats. For the winter data, we summed the hourly energy expenditure predicted by the regression for the mean burrow temperature and the mean *T*_e_ for the entire month. We followed the same procedure for estimating the energy expenditure in the burrow for one month during summer, but because pygmy rabbits demonstrated high capacity for behavioral thermoregulation through above-ground rest site selection during summer (Milling et al., 2017), we used the lowest measured hourly *T*_e_ in the regression for instances when the mean *T*_e_ exceeded the estimated *T*_uc_ of pygmy rabbits. To estimate energy expenditure above 35 °C during summer, we assumed that $\dot V{{\rm{O}}_2}$ increased at the same rate above the TNZ as it did with increasing cold below the TNZ, which is an assumption that is supported in at least two other species of leporid (Hinds, 1973, 1977). Because model uncertainty propagates to the predicted estimates of thermoregulatory costs, we used a simulation-based approach described by Mandel (2013) to estimate the standard errors of the expected cost for each season and microhabitat using the measured temperature values. This method relies on a parametric bootstrap approach using the estimated sampling distribution of the model parameters instead of analytical or numerical derivatives.

## Results

### Thermal physiology

We measured $\dot V{{\rm{O}}_2}$ for six animals in winter (four females and two males) and six animals in summer (four females and two males). Three animals were used in both season, and so our analysis reflects a sample size of nine individuals (six females and three males). One animal died before completion of the trials, so $\dot V{{\rm{O}}_2}$ for that individual was only measured at five temperatures during winter. Additionally, we eliminated 5 °C data from two animals (one in summer and one in winter) because they were active during measurements, and we were unable to determine resting $\dot V{{\rm{O}}_2}$. The final dataset included 74 trials (35 in summer and 39 in winter). Approximately 20% of the variance in the data was attributable to the random effect of individual (${s^2}_{{\!\!\!\rm{animal}}} = 0.33,\;{s^2}_{{\!\!\!\rm{resid}}} = 1.38,\;{s^2}_{{\!\!\!\rm{total}}} = 1.70$). Animals averaged 462 ± 42.2 g, and mass was positively correlated with RMR (*p* = 0.041).

**Figure 2:**
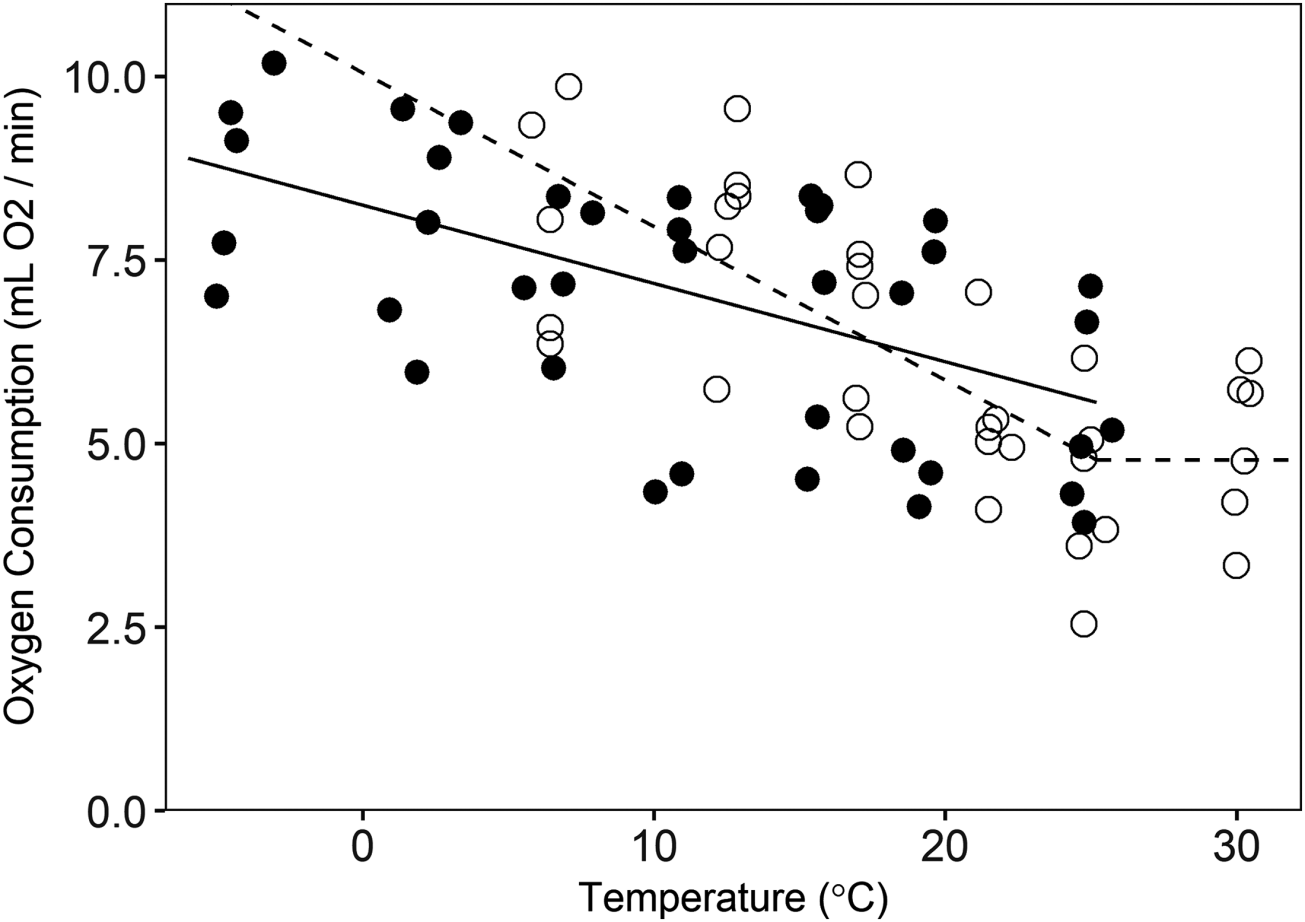
Oxygen consumption by pygmy rabbits (*Brachylagus idahoensis*) at different temperatures during summer (open circles, dashed line) and winter (filled circles, solid line). The line segments at temperatures below the breakpoint illustrate the relationship between oxygen consumption and temperature below the lower critical temperature, whereas the line segment above the breakpoint shows minimal resting metabolic rate in the thermoneutral zone of summer-acclimatized animals.

Season had a significant effect (*p* = 0.017) on the slope of the $\dot V{{\rm{O}}_2}$ versus temperature regression below the *T*_lc_. During summer, the slope was −0.21 ± 0.04 mL O_2_ min^−1^ °C^−1^ (*p* < 0.001), but in winter the slope was −0.11 ± 0.02 mL O_2_ min^−1^ °C^−1^ (*p* < 0.001; Fig. 2). This difference equates to 22% higher thermoregulatory costs at 0 °C in summer than in winter for an average size animal, with the magnitude of the seasonal cost difference decreasing with increasing temperatures (Fig. 2). Based on visual inspection, the temperatures for our winter trials did not appear to exceed *T*_lc_, and therefore, we could not estimate winter values for this parameter or RMR_T_. The *T*_lc_ was estimated at 25.2 ± 2.9 °C, and we assumed a consistent value across seasons (we could not test for an effect of season on this parameter, but there was no difference in the value of *T*_lc_ when the model was estimated for summer data alone (not presented here) versus summer and winter data combined). Summer RMR_T_ was 4.78 ± 0.51 mL O_2_/min for an animal of average size. We did not detect the *T*_uc_ in either summer or winter (our test temperatures were not high enough to elicit increased RMR, hence the use of a literature-supported estimate of the *T*_uc_), but we did observe differing postures between high and low trial temperatures. During warmer trials (i.e., 25 and 30 °C), animals extended their bodies and assumed a sprawled posture, presumably to maximize contact with the chamber floor. This contrasted with the typical spherical posture during cooler trials. The mean values of thermal conductance for summer- and winter-acclimatized animals was 15.14 ± 1.35 mL O_2_ h^−1^ °C^−1^ and 12.28 ± 0.91 mL O_2_ h^−1^ °C^−1^, respectively (mass-adjusted thermal conductance in summer = 0.033 ± 0.003 mL O_2_ h^−1^ g^−1^ °C^−1^ and winter = 0.027 ± 0.004 mL O_2_ h^−1^ g^−1^ °C^−1^). There was no significant difference between summer and winter thermal conductance (*t* = −1.7, CI = −6.0–0.2), nor was there a significant effect of mass on thermal conductance (*t* = 0.47, CI = −0.06–0.04).

### Microsite temperature

**Figure 3:**
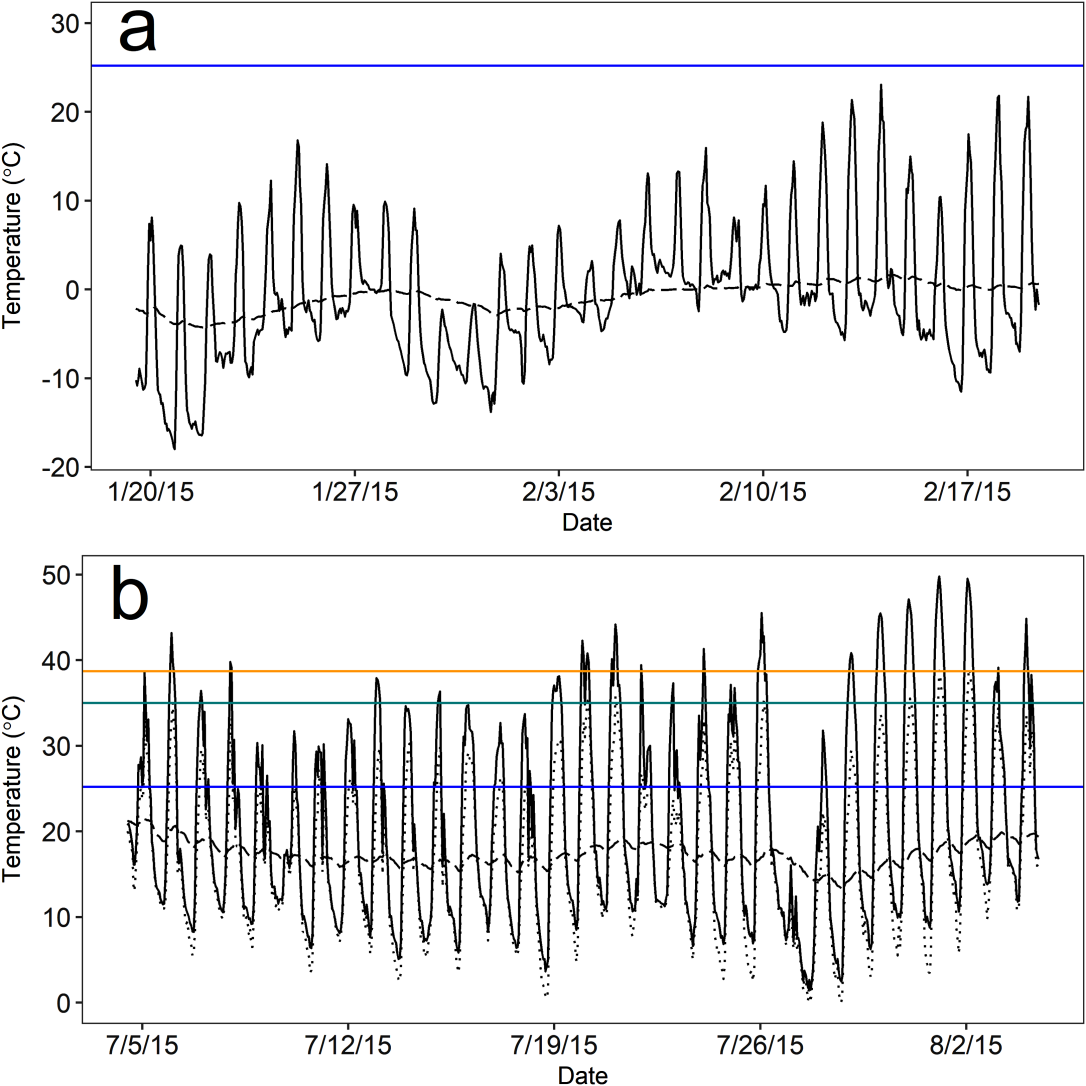
Mean operative temperature (*T*_e_) at above-ground microsites (solid black line) and temperatures within pygmy rabbit (*Brachylagus idahoensis*) burrows (dashed black line). Temperature was measured during the (A) winter (20 January–19 February, 2015) and (B) summer (5 July–4 August, 2015) in east-central Idaho, USA. Mean temperatures above and below ground remained below the lower critical temperature (blue line) for the duration of winter monitoring. Mean above-ground *T*_e_ often exceeded the estimated upper critical temperature (green line) and body temperature (orange line) of pygmy rabbits during summer, but there were typically above-ground rest sites available that were cooler than the mean (lowest hourly *T*_e_ measured by a single operative temperature model; dotted black line).

As expected, the thermal environment (as *T*_e_) of above-ground rest sites was more extreme and variable than the burrow thermal environment during both seasons, but burrows provided more stable microclimates during winter than summer based on visual inspection. Notably, mean temperatures both above ground and in burrows remained below the estimated *T*_lc_ (25.2 °C) of pygmy rabbits for nearly the entire duration of winter monitoring. During winter, mean hourly *T*_e_ ranged from −18.0 to 23.1 °C, and burrow temperatures ranged from −4.3 to 1.7 °C (Fig. 3). Operative temperatures only exceeded the estimated *T*_lc_ at a monitored, above-ground site on eight out of 31 days and never remained above the *T*_lc_ for more than five continuous hours (mean = 2.2 h, range = 1–5 h). Mean hourly *T*_e_ in summer ranged from 1.5 to 49.8 °C (Fig. 3). However, the minimum hourly *T*_e_ reported by a single device ranged from 0 to 39 °C. Summer burrow temperatures were cooler than daily high *T*_e_ values and less variable, ranging from 13.3 to 21.4 °C (Fig. 3), which remained below the *T*_lc_ of our pygmy rabbits.

The burrow satisfied our definition of thermal refuge for a greater proportion of time during winter than summer. Burrows were thermal refuges on 30 of the 31 days that we monitored temperature during the winter, for an average of 13.4 h per day (sd = 5.5; 55.8% of a 24 h day). Burrows were generally warmer than *T*_e_ during the overnight and early morning periods, whereas above-ground microsites were warmer than burrows from approximately 0900 to 1800 h. During summer, burrows were thermal refuges on 21 of 31 days for an average of 5.0 h per day (sd = 3.0; 20.6% of a 24 h day). Accounting for the lowest mean temperature measured by a single *T*_e_ sensor in a given hour, the number of days in which *T*_e_ exceeded 35 °C (the estimated *T*_uc_) dropped to six days, and *T*_e_ remained above 35 °C for 2.5 h per day (sd = 2.0; 10.4% of a 24 h day). High temperatures occurred during mid-day (1000–1700 h), likely as a result of solar radiation. During these periods, burrow temperatures averaged 17.7 °C (sd = 1.5) and were 22.5 °C cooler than *T*_e_, suggesting that burrow use could reduce the energy and water costs of thermoregulation when *T*_e_ is considerably greater than *T*_uc_.

Although the thermal environment at our study site was variable and was outside of the TNZ of pygmy rabbits during both seasons, the variety of microclimates available provided opportunities to reduce costs of thermoregulation. The thermoregulatory costs associated with using a burrow as a thermal refuge during winter were lower than using only above-ground or burrow microhabitats exclusively (Table 1). During summer, however, predicted thermoregulatory costs associated with resting above ground exclusively were no different than using a burrow as a thermal refuge during brief periods of high *T*_e_ because the burrow temperatures often were below *T*_lc_ (Table 1). The heterogeneity present in above-ground rest sites provided microclimates that reduced the need for regulatory evaporative cooling and imposed lower thermoregulatory costs than would be incurred by resting inside the burrow.

**Table 1:** Predicted thermoregulatory costs (±SE) incurred by pygmy rabbits (*Brachylagus idahoensis*) for one month in summer and winter 2015 in different microhabitats in east-central Idaho, USA.

Microhabitat	Winter thermoregulatory costs (kJ)	Summer thermoregulatory costs (kJ)^†^
Above ground only	7460.7 (331.4)	5724.7 (274.0)
Burrow only	7479.1 (340.2)	5770.1 (295.2)
Above ground + burrow refuge^‡^	7211.2 (318.2)	5745.2 (283.2)

**Notes:**

† The coolest available above-ground microsites were used to calculate energy expenditure above the upper critical temperature during summer.

‡ Burrows were considered to be thermal refuge in summer when above-ground mean operative temperature (*T*_e_) > 35 °C and in the winter when the burrow temperature was warmer than *T*_e_.

## Discussion

Pygmy rabbits at our study site often were exposed to thermal conditions outside of their TNZ, but we documented seasonal differences in the relationship between temperature and energy expenditure suggesting that rabbits acclimatized to prevailing thermal conditions. Availability of diverse thermal microsites likely reduced the energy costs of thermoregulation, and burrows provided refuge from extreme, above-ground temperatures during both seasons. This buffering was especially important during winter, when both *T*_e_ and burrow temperatures were below the *T*_lc_, but the estimated cost of thermoregulation in burrows was lower than above ground. Availability of sheltered microsites above ground during summer resulted in relatively short periods when *T*_e_ > 35 °C across the landscape, reducing the energetic advantages to using a burrow during that season. This work supports the notion that selective use of burrows can be an effective strategy for mitigating the thermoregulatory costs of inhabiting a strongly seasonal environment.

We documented a seasonal change in the relationship between temperature and $\dot V{{\rm{O}}_2}$ below the *T*_lc_ that resulted in enhanced energy conservation in cold winter conditions while facilitating heat loss at high summer temperatures. At *T*_e_ = 0 °C, estimated thermoregulatory costs were 22% higher during summer than winter. A similar relationship has been observed in a variety of other cold-acclimatized endotherms and is an important adaptive strategy for inhabiting cold climates (Hinds, 1973, 1977; Swanson, 1991; Holloway & Geiser, 2001). Additionally, pygmy rabbits have lower thermal conductance during winter than predicted based on their body size (predicted mass-adjusted thermal conductance = 0.0557 mL O_2_ h^−1^ g^−1^ °C^−1^ for a 462 g animal; Bradley & Deavers, 1980; Katzner, Parker & Harlow, 1997), which would enhance energy conservation under cold temperatures, even in the absence of other winter-acclimatization strategies. Notably, we observed identical values of thermal conductance in winter-acclimatized pygmy rabbits as those reported by Katzner, Parker & Harlow (1997). Collectively, these results suggest that pygmy rabbits possess important physiological adaptations that allow them to persist in unfavorable winter climates.

The RMR_T_ that we measured in summer-acclimatized animals, 4.78 mL O_2_/min, is similar to the expected basal metabolic rate (BMR) for a 462 g eutherian mammal (4.94 mL O_2_/min, Hayssen & Lacy, 1985; 4.65 mL O_2_/min, White & Seymour, 2004), but lower than predicted for other lagomorphs (7.00 mL O_2_/min; Hayssen & Lacy, 1985). This value also is lower than previously reported values of RMR_T_ of winter-acclimatized pygmy rabbits (6.85 mL O_2_/min for a 462 g animal; Katzner, Parker & Harlow, 1997). Intraspecific variation in RMR_T_ can be substantial (Bech, Langseth & Gabrielsen, 1999; Speakman, Krol & Johnson, 2004) and can be a function of differences in individual personality (Careau et al., 2008), diet quality (Rosen & Trites, 1999), or local adaptation (Mathias et al., 2006). Indeed, different studies have identified dissimilar RMR_T_ values for American pikas (*Ochotona princeps*; MacArthur & Wang, 1973; Otto, Wilson & Beever, 2015) and North American porcupines (*Erethizon dorsatum*; DeMatteo & Harlow, 1997; Fournier & Thomas, 1999). Our values of RMR_T_ might differ from those documented by Katzner, Parker & Harlow (1997) because of differences in body size between our studies (our animals were slightly larger), differences in husbandry, acclimatization to different environmental conditions (summer versus winter), or population-level differences in RMR_T_. Although we were not able to determine the RMR_T_ of winter-acclimatized animals, the convergence of the summer and winter regressions on similar values of RMR_T_ at the *T*_lc_ (Fig. 2) suggested that a seasonal difference in RMR_T_ of pygmy rabbits is unlikely.

Although we could not quantify a shift in *T*_lc_ in winter relative to summer, the data suggested that the difference between seasons was minimal. Small endotherms often are limited in their capacity to add insulation via fat deposits or thicker winter pelage, and *T*_lc_ fluctuates very little, if at all, as a result (McNab, 2002; Marchand, 2013). Although a seasonal shift in *T*_lc_ has been documented for several larger-bodied, non-burrowing leporids (*Sylvilagus audobonii—*Hinds, 1973; *Lepus alleni* and *L. californicus—*Hinds, 1977; *L. townsendii—*Rogowitz, 1990; *L. timidus—*Pyornila et al., 1992; *L. americanus—*Sheriff et al., 2009), constancy of *T*_lc_ between seasons has been documented in several small endotherms including red squirrels (Irving, Krog & Monson, 1955), black-capped chickadees (Cooper & Swanson, 1994), greenfinches (*Carduelis chloris*; Saarela, Klapper & Heldmaier, 1995), and dark-eyed juncos (*Junco hyemalis*; Swanson, 1991). Use of thermal refuges has an important influence on thermal physiology (Jackson et al., 2002). Indeed, Jackson, Bennett & Spinks (2004) documented similar thermophysiological characteristics (RMR_T_, *T*_lc_, and *C*) among species of Otomyinae across a mesic-arid gradient, and suggested that these properties reflected similar refuge strategies rather than prevailing environmental conditions. The ability of pygmy rabbits to use burrows may contribute to seasonal constancy of *T*_lc_ and RMR_T_ by allowing the animal to use behavioral means of increasing thermal resistance rather than physiological acclimatization alone. Exploring relationships between the use of thermal refuges and seasonal morphological and physiological acclimatization in pygmy rabbits remains a fruitful area for future research.

Our data showed that burrows provide important thermal refuge for pygmy rabbits during winter by reducing the energetic costs of thermoregulation relative to above-ground microsites. Winter temperatures at our study site rarely warmed to the TNZ of pygmy rabbits in either microhabitat, but burrows provided buffered microclimates and imposed lower thermoregulatory costs than above-ground rest sites for more than 50% of the monitoring period. Our observations of the role of the burrow as thermal refuge are consistent with the behavior of pygmy rabbits during winter. Lee et al. (2010) documented rabbits near burrow openings more frequently during winter than during summer or autumn. Milling et al. (2017) noted reductions in winter activity levels with cold temperatures during the night and early morning, and proximity of a burrow was the dominant factor in rest site selection by pygmy rabbits during winter, but not summer. Although proximity to refuge might reflect a heightened perception of predation risk (Crowell et al., 2016), our results suggested that thermal risk might also account for these behaviors during winter.

Burrows do not seem to be as critical for thermal refuge for pygmy rabbits during summer. In fact, we predicted similar thermoregulatory costs in both above- and below-ground rest sites. Although average *T*_e_ at the study site was typically above estimated *T*_uc_ for several hours daily, *T*_e_ at some above-ground microsites was considerably lower and within the TNZ throughout the day because of the shade provided by dense overhead canopy. Shaded microsites and cool soil would negate the need to seek thermal refuge inside a burrow for an animal at rest. During the brief periods when above-ground temperatures exceeded the TNZ, the cost of cooling in the sheltered locations was predicted to be lower than the cost of warming inside the burrow at the same time because the difference in temperature between the burrow and *T*_lc_ was greater than that between the *T*_uc_ and *T*_e_. Piute ground squirrels (*Spermophilus mollis*) relied on burrows less in sagebrush steppe than in grassland habitats because the structural complexity of sagebrush offered more thermal heterogeneity and suitable microclimates: instead of using burrows for cooling, ground squirrels stretched out on the ground in the shade (Sharpe & Van Horne, 1999). We observed similar behavior by free-ranging pygmy rabbits: animals were repeatedly found resting in shallow depressions in the soil (i.e., forms) in the shade, presumably as a behavioral thermoregulation strategy (Milling et al., 2017). Alternatively, burrows may be an important resource for “shuttling” thermoregulation (i.e., moving between patches of optimal and sub-optimal thermal conditions to exploit different resources) that behaviorally ameliorates energy and water costs during periods of very high temperatures (Chappell & Bartholomew, 1981; Vispo & Bakken, 1993; Hainsworth, 1995). The energetic advantages of such fine-scale behavior cannot be quantified in the present study but remains a compelling hypothesis for future investigation.

Actual energy expenditure during both seasons likely differs from our estimates in several ways. We suspect that our estimate of thermoregulatory savings from burrow use during winter is conservative because *T*_e_ does not incorporate wind-induced reductions in thermal resistance that can greatly increase heat loss (i.e., “wind chill factors”; Bakken, 1980). Rogowitz & Gessaman (1990) documented an interactive effect between wind and temperature on metabolic rate of white-tailed jackrabbits (*Lepus townsendii*) during the winter, such that exposure to wind radically elevated metabolic rate at low temperatures. Thus, the thermoregulatory cost savings of using a burrow as a thermal refuge during the winter is potentially larger than our model predicts. Even given the uncertainty associated with the propagation of model error on thermoregulatory costs in each of the microhabitats, we believe that our results represent the relative utility of the burrow as a thermal refuge when above-ground climatic conditions are inhospitable. During the summer, the lowest *T*_e_ values on our study site occurred during the night and early morning and were colder than the burrow and below the *T*_lc_. Pygmy rabbits are active through the night and crepuscular periods during the summer (Milling et al., 2017) and are likely capable of substituting some heat produced during locomotion (exercise thermogenesis) for regulatory heat production (Humphries & Careau, 2011). Our estimates of energy expenditure are for resting animals, but heat generated during activity might explain why extensive overnight use of burrows during summer has not been observed (Lee et al., 2010). Similarly, our estimates of energy expenditure in above-ground rest sites are likely conservative. The relationship between energy expenditure and temperature above the *T*_uc_ can be steeper than the relationship below the *T*_lc_ because the process of evaporative cooling itself produces heat (Humphries & Umbanhowar, 2007). Because we were not able to measure RMR above the *T*_uc_, the true thermoregulatory costs associated with resting above ground at high *T*_e_ could include an unknown increment of metabolic rate from active evaporative cooling (panting, salivation, etc.). Furthermore, our estimates of energy expenditure do not address evaporative water loss, which is likely also an important factor in the thermal physiology and overall fitness of this species. Nonetheless, because pygmy rabbits demonstrated a high capacity for behavioral thermoregulation (Milling et al., 2017), the burrow likely did not confer considerable thermoregulatory cost savings over selection of thermally suitable above-ground rest sites.

Burrow use also is influenced by other factors besides behavioral thermoregulation including reproduction and predator avoidance (Rachlow, Sanchez & Estes-Zumpf, 2005; Elias et al., 2006; Camp et al., 2012). Additionally, co-occurring species, such as ground squirrels, weasels, other leporids, reptiles and invertebrates use pygmy rabbit burrows (Green & Flinders, 1980; Lee et al., 2010), and it is unclear how these interactions might influence burrow use by pygmy rabbits. Some predators on our study site, such as badgers (*Taxidea taxus*) and long-tailed weasels (*Mustela frenata*), are capable of retrieving a pygmy rabbit from a burrow system (Oliver, 2004), and vigilance and detection would be impaired for an animal at rest in a burrow. Our estimation of the thermoregulatory costs associated with resting above and below ground do not allow us to explicitly test hypotheses regarding the specific circumstances under which pygmy rabbits would use burrow systems, but they do provide compelling support for the functional role of a burrow as thermal refuge for the species and how that role might change between seasons.

## Conclusion

Microhabitat selection and its influence on physiology can have important ramifications for individual fitness (Huey, 1991). Our research suggests that pygmy rabbits acclimatize seasonally by reducing energy expenditure at cold temperatures in winter relative to summer, and they have lower than expected thermal conductance. These qualities confer important thermoregulatory cost savings during the winter. Even so, the burrow is an important thermal refuge, particularly in winter when thermoregulatory costs can be reduced by resting in thermally buffered microsites below ground. However, reductions in snow cover are associated with increased thermoregulatory costs for burrow users (Geiser & Turbill, 2009) and are predicted under most climate change scenarios (Pauli et al., 2013). Although substantial efforts have focused on effects of climate change-induced shifts in precipitation and temperature on hot-acclimated animals, associated changes in winter ecology may have greater implications for individual fitness and population persistence for animals such as pygmy rabbits (Pauli et al., 2013; Williams, Henry & Sinclair, 2014). Climate and land-use changes in the future will undoubtedly continue to modify the thermal environment for numerous species through changes to vegetation composition and structure and shifts in large-scale weather patterns. Understanding the extent to which such changes can influence the value of below-ground refuges, however, begins with understanding the functional relationship between the physiology of an organism and the microhabitats it exploits.

## Supplemental Information

10.7717/peerj.4511/supp-1 Supplemental Information 1 Data and R-code for performing non-linear mixed effects segmented regression on respirometry data.

10.7717/peerj.4511/supp-2 Supplemental Information 2 Environmental temperatures and associated thermoregulatory costs for above- and below-ground microhabitat in the sagebrush steppe.

10.7717/peerj.4511/supp-3 Supplemental Information 3 Metadata (abbreviations and descriptions) for Milling_etal_EnviroTemps.csv and Milling_etal_nlmePYRA.R.

10.7717/peerj.4511/supp-4 Supplemental Information 4 Temperatures for predicting thermoregulatory costs in each microhabitat and estimating standard errors. This dataset is required to perform the bootstrapping procedure to estimate standard errors of resting metabolic rate.

## Additional Information and Declarations

### Field Study Permissions

The following information was supplied relating to field study approvals (i.e., approving body and any reference numbers): Scientific Collection Permits for collection of adult pygmy rabbits were obtained from Idaho Department of Fish and Game and Montana Fish, Wildlife, and Parks.

### Data Deposition

The following information was supplied regarding data availability: The files containing raw data and code are provided as Supplemental Dataset Files.
